# Feline coronavirus with and without spike gene mutations detected by real-time RT-PCRs in cats with feline infectious peritonitis

**DOI:** 10.1177/1098612X19886671

**Published:** 2019-11-15

**Authors:** Laura Emmler, Sandra Felten, Kaspar Matiasek, Hans-Joerg Balzer, Nikola Pantchev, Christian Leutenegger, Katrin Hartmann

**Affiliations:** 1 Clinic of Small Animal Medicine, Centre for Clinical Veterinary Medicine, Ludwig Maximilian University of Munich, Munich, Germany; 2 Section of Clinical and Comparative Neuropathology, Institute of Veterinary Pathology, Centre for Clinical Veterinary Medicine, Ludwig Maximilian University of Munich, Munich, Germany; 3 IDEXX Laboratories, Ludwigsburg, Germany; 4 IDEXX Laboratories, West Sacramento, CA, USA

**Keywords:** FCoV, FIP, RT-PCR, S gene, IHC, immunohistochemistry

## Abstract

**Objectives:**

Feline infectious peritonitis (FIP) emerges when feline coronaviruses (FCoVs) mutate within their host to a highly virulent biotype and the immune response is not able to control the infection. FCoV spike (*S*) gene mutations are considered to contribute to the change in virulence by enabling FCoV infection of and replication in macrophages. This study investigated the presence of FCoV with and without *S* gene mutations in cats with FIP using two different real-time RT-PCRs on different samples obtained under clinical conditions.

**Methods:**

Fine-needle aspirates (FNAs) and incisional biopsies (IBs) of popliteal and mesenteric lymph nodes, liver, spleen, omentum and kidneys (each n = 20), EDTA blood (n = 13), buffy coat smears (n = 13), serum (n = 11), effusion (n = 14), cerebrospinal fluid (n = 16), aqueous humour (n = 20) and peritoneal lavage (n = 6) were obtained from 20 cats with FIP diagnosed by immunohistochemistry. Samples were examined by RT-PCR targeting the FCoV *7b* gene, detecting all FCoV, and *S* gene mutation RT-PCR targeting mutations in nucleotides 23531 and 23537. The prevalence of FCoV detected in each sample type was calculated.

**Results:**

In 20/20 cats, FCoV with *S* gene mutations was present in at least one sample, but there was variation in which sample was positive. FCoV with mutations in the *S* gene were most frequently found in effusion (64%, 95% confidence interval [CI] 39–89), followed by spleen, omentum and kidney IBs (50%, 95% CI 28–72), mesenteric lymph node IBs and FNAs (45%, 95% CI 23–67), and FNAs of spleen and liver and liver IBs (40%, 95% CI 19–62).

**Conclusions and relevance:**

In these 20 cats with FIP, FCoVs with *S* gene mutations were found in every cat in at least one tissue or fluid sample. This highlights the association between mutated *S* gene and systemic FCoV spread. Examining a combination of different samples increased the probability of finding FCoV with the mutated *S* gene.

## Introduction

Feline infectious peritonitis (FIP) is one of the most important infectious diseases in cats, but its pathophysiology is still not fully understood. According to the internal mutation theory, FIP emerges when feline coronaviruses (FCoVs) mutate within their host to a highly virulent biotype^1,2^ and the host’s immune system is not able to control the infection.^3,4^

The exact nature of mutations that are responsible for the development of FIP is not known yet. A combination of different mutations on different genes is likely as mutations that have been identified to date do not qualify as sole causes for FIP.^5–8^ This results in FCoV strains with different genome sequences in each cat with FIP,^6,9,10^ highlighting that there are multiple pseudo-strains of FCoV within an individual cat and that a single consistent mutation responsible for all cases of FIP does not exist. Following mutation, increased virulence of FCoV is the result of a change in viral cell tropism from enterocytes to macrophages and efficient replication within these cells.^11,12^ As the FCoV spike (S) protein plays a key role in viral cell entry,^13^ studies have investigated the mutations in the *S* gene as possible contributing reasons for the change in virulence.^14–16^ One study identified mutations in close proximity in the *S* gene’s nucleotides 23531 and 23537, causing two different amino acid substitutions in the S protein.^5^ In contrast to other *S* gene mutations,^14^ mutations in nucleotide 23531 and 23537 were identified in 96% of FCoVs isolated from cats with FIP in that study. These mutations were not identified in faecal samples of clinically healthy control cats in that study; however, no organ samples from these control cats were analysed.^5^

Immunological staining of viral antigen within tissue lesions is considered the reference standard for diagnosing FIP,^17–19^ but it requires invasive sampling. Molecular methods, such as real-time RT-PCR, have evolved in the past years. RT-PCR detecting FCoV is only partially useful,^20–22^ as viral RNA also circulates within asymptomatic FCoV-infected cats not suffering from FIP.^20,23,24^ Detection of the abovementioned FCoV *S* gene mutations^5^ might help in the diagnosis of FIP as studies examining detection of these *S* gene mutations via RT-PCR and/or pyrosequencing confirmed that these mutations are present in the majority of cats with FIP.^25–27^ However, the same mutations were also detected in cats without FIP.^28,29^ Therefore, the presence or detection of FCoV with *S* gene mutations in samples does not automatically equate to the presence of FIP. Sensitivity and specificity of diagnosing FIP by detecting these mutations in specific fluids (eg, serum or effusion) and tissue samples have already been investigated,^25–29^ but only a few studies compared different sample types.

The present study investigated 20 cats with FIP confirmed by tissue immunohistochemistry (IHC). The study aimed to evaluate the presence of FCoV with and without *S* gene mutations in a variety of different tissue and fluid samples that can be obtained under clinical conditions. Methods used were two different RT-PCRs using primers to detect all FCoV (*7b* gene RT-PCR) and primers detecting *S* gene mutations in nucleotides 23531 and 23537 (*S* gene mutation RT-PCR).

## Materials and methods

### Cats

Twenty cats were prospectively included (Table 1). All cats were presented for suspected FIP from 2015 to 2017 and were euthanased owing to poor general condition. FIP was confirmed by histopathology and immunostaining of FCoV antigen in tissue macrophages in all 20 cats. Only cats with positive IHC were included. IHC was performed using clone FIPV3-70 antibody (Linaris Medizinische Produkte GmbH) on formalin-fixed, paraffin-embedded tissue sections.^30^ For signal detection, the streptavidin–biotin complex method was implemented (VECTASTAIN ABC Kit; Vector Laboratories). Negative controls were included in which the antibody was substituted by phosphate buffered saline (PBS). Samples were considered as positive if typical histological lesions were present (eg, granulomatous vasculitis or granulomatous inflammation in tissues) and FCoV antigen was detected in macrophages in those lesions. Tissues with positive IHC results are listed in Table 1.

**Table 1:** Cats with feline infectious peritonitis (FIP) included in the study

Cat	Breed	Sex	Age	Effusion	Neurological or ocular signs	Tissues with FIP-typical lesions and positive IHC
1	DSH	MI	10 mo	Yes	Neurological and ocular signs	Liver, spleen, kidneys, mesenteric lymph nodes
2	DSH	MN	1.5 y	Yes	No	Kidneys, omentum
3	DSH	MN	3 y	No	No	Spleen, omentum
4	Birman	MN	2.5 y	No	No	Kidneys, mesenteric lymph nodes
5	Birman	FI	7 mo	No	No	Liver, kidneys, mesenteric lymph nodes
6	DSH	MN	7 y	Yes	No	Liver, spleen, kidneys, mesenteric lymph nodes, omentum
7	DSH	FI	1 y	Yes	No	Mesenteric lymph nodes
8	DSH	FI	5 mo	Yes	No	Mesenteric lymph nodes, omentum
9	DSH	MI	2 y	Yes	No	Liver, spleen, kidneys, mesenteric lymph nodes, omentum
10	DSH	FI	6 mo	Yes	Neurological signs	Liver, omentum
11	DSH	MI	7 mo	Yes	No	Liver, spleen, mesenteric lymph nodes, omentum
12	DSH	MI	3 y	Yes	No	Liver, spleen, mesenteric lymph nodes, omentum
13	Persian	FI	1.5 y	No	No	Mesenteric lymph nodes
14	DSH	FI	1.5 y	Yes	No	Spleen
15	DSH	MN	6 y	No	No	Spleen, kidneys, mesenteric lymph nodes, omentum
16	DSH	FI	5 mo	Yes	No	Spleen, kidneys, mesenteric lymph nodes, omentum
17	DSH	MI	9 mo	Yes	No	Liver, spleen, mesenteric lymph nodes, omentum
18	Mix	FI	6 mo	No	Ocular signs	Spleen, kidneys, mesenteric lymph nodes, omentum
19	DSH	MN	10 mo	Yes	No	Mesenteric lymph nodes, omentum
20	DSH	MN	14 y	Yes	No	Mesenteric lymph nodes, omentum

IHC = immunohistochemistry; DSH = domestic shorthair; MI = male intact; MN = male neutered; FI = female intact; mo = months; y = years

Blood samples (EDTA blood, buffy coat smear, serum) were obtained ante mortem for diagnostic purposes in all cats. Effusion was obtained ante mortem for diagnostic and therapeutic purposes. Cerebrospinal fluid (CSF) and aqueous humour were obtained by paracentesis directly after euthanasia. Peritoneal lavage was performed post mortem with 20 ml/kg sodium chloride solution (0.9%) in cats that did not have effusion. Fine-needle aspirates (FNAs) and incisional biopsies (IBs) of all organs were obtained post mortem during necropsy, independently of the presence of lesions. IBs were stored in Eppendorf tubes with sodium chloride solution (0.9%). FNAs were layered on slides without staining. All samples were stored at 4°C until shipping. Refrigeration has no impact on RNA degradation but was performed for logistic reasons. Shipping was performed without refrigeration. Time between sampling and examination never exceeded 72 h.

### RT-PCRs

RT-PCRs were performed at a commercial laboratory (IDEXX Laboratories, Ludwigsburg, Germany). RT-PCRs were performed with six quality controls. Extraction of total nucleic acid (TNA) was performed using QIAamp DNA Blood BioRobot MDx Kit on an automated Qiagen platform, according to the manufacturer’s instructions. TNA was extracted from 200 µl of any kind of liquid diagnostic sample. EDTA blood and serum were applied without prior treatment following the extraction protocol of the manufacturer. Effusion, CSF, aqueous humour and peritoneal lavage samples were centrifuged and the sediment resuspended in 200 µl of remaining sample fluid introduced into the extraction procedure. Clinical material on slides was dissolved with 200 µl of PBS and the obtained suspension was used for TNA extraction.

In the case of tissue samples, 20 mg was pretreated with Proteinase K according to the manufacturer’s protocol. Firstly, the *7b* gene RT-PCR targeting FCoV *7b* gene was performed to quantify the viral load.^31^ Secondly, the two RT-PCRs were performed targeting the M1058L and S1060A single nucleotide polymorphisms (SNPs) within the fusion peptide of the S protein (IDEXX Laboratories, unpublished data). The *S* gene mutation RT-PCRs allow the typing of an FCoV strain based on the presence or absence of one of two SNPs within the fusion peptide of the *S* gene. The paired *S* gene mutation RT-PCRs were previously validated analytically using synthetic DNA positive controls (IDT DNA), as well as clinically using samples collected from cats originally used to identify the two *S* gene mutations: (1) FCoV-infected and shedding, but otherwise healthy; and (2) affected by FIP.^32^

Additional studies have evaluated RT-PCR detection of FCoV mutations in paraffin-embedded tissues and effusion from cats with confirmed FIP.^27,33^ Briefly, highly specific hydrolysis probes were used, detecting either the mutation at position 3174 (A → C/T) or 3180 (T → G) on the FCoV genome, corresponding to amino acid positions 1058 and 1060, nucleotide 23531 and 23537, and M1058L and S1060A of reference sequence FJ938051, respectively, or non-mutated sequences by using an allelic discrimination approach (IDEXX Laboratories, unpublished data). Probes for mutated and non-mutated *S* gene sequences were fluorophore-labelled (6-FAM and VIC, respectively). Results were analysed detecting the 6-FAM:VIC (mutated:non-mutated) fluorescence ratio emitted by the hydrolysis probes.

*S* gene mutation RT-PCR was considered positive for either mutation when fluorescence in the mutation probe was at least two-fold higher than in the non-mutated probe. *S* gene mutation RT-PCR was classified as negative if: (1) no FCoV was detected; (2) FCoV without one of the two *S* gene mutations was detected; (3) FCoV load was below the cut-off of 1.5 million RNA equivalents per ml, which did not allow a successful differentiation of the FCoV strains via *S* gene mutation RT-PCR; or (4) no further differentiation via *S* gene mutation RT-PCR was possible despite a high FCoV load (above 1.5 million RNA equivalents per ml of sample). *S* gene mutation RT-PCR was considered as positive if: (1) FCoV with a mutated *S* gene (either mutation in nucleotide 23531 or 23537); or (2) both mutated and non-mutated *S* genes were detected in the same sample.

### Data analysis

The prevalance of positive results for *7b* gene RT-PCR and *S* gene mutation RT-PCRs in different tissues and body fluids were calculated by dividing the number of positive results by the total number of examined samples of that specific tissue or fluid. Ninety-five per cent confidence intervals (CIs) were calculated.

## Results

FCoV with a mutated *S* gene was detected in all 20 cats in at least one tissue or fluid. The type of samples with a positive *S* gene mutation RT-PCR result differed from cat to cat (Tables 2 and 3). The prevalence of FCoV with and without a mutated *S* gene detected by RT-PCR in each tissue and fluid are listed in Table 4. *S* gene mutation RT-PCR was less commonly positive than *7b* gene RT-PCR. *S* gene mutation RT-PCR was most commonly positive in effusion (64.3%). Serum samples and buffy coat smears showed no positive results for *S* gene mutation RT-PCR in any cats. The percentages of positive results of both RT-PCRs were similar or even identical for FNAs and IBs in intra-abdominal organs. All samples positive in *S* gene mutation RT-PCRs had the mutation in nucleotide 23531; in none of the examined samples was a mutation in nucleotide 23537 present.

**Table 2:** Results of *7b* gene and *spike* gene mutation RT-PCRs in different tissues

Cat	Sample	Popliteal lymph node	Mesenteric lymph node	Liver	Spleen	Omentum	Kidneys
1	FNA	M1058L*	Neg^†^	M1058L	Low^‡^	–	–
	IB	Low	Low	Low	Low	M1058L	M1058L
2	FNA	Low	M1058L	Low	M1058L	–	–
	IB	Low	M1058L	Low	Low	M1058L	Low
3	FNA	Neg	M1058L	Neg	Neg	–	–
	IB	Neg	M1058L	Neg	Neg	Neg	Neg
4	FNA	Low	Neg	Low	Low	–	–
	IB	Mixed FCoV^§^	Neg	Neg	Neg	Low	Mixed FCoV
5	FNA	Low	Low	Low	Low	–	–
	IB	Neg	Low	M1058L	Low	Low	Low
6	FNA	Neg	Low	M1058L	M1058L	–	–
	IB	M1058L	M1058L	M1058L	M1058L	M1058L	M1058L
7	FNA	Low	Low	Low	Low	–	–
	IB	Neg	Non-mutated *S* gene^¶^	Low	Low	Low	Neg
8	FNA	Low	High^∞^	High	High	–	–
	IB	Low	High	Low	M1058L	M1058L	Mixed FCoV
9	FNA	M1058L	M1058L	M1058L	M1058L	–	–
	IB	Low	M1058L	Mixed FCoV	Mixed FCoV	M1058L	Low
10	FNA	Low	Low	Low	Low	–	–
	IB	M1058L	High	Low	High	Low	M1058L
11	FNA	Neg	M1058L	Mixed FCoV	M1058L	–	–
	IB	Neg	M1058L	M1058L	M1058L	Low	M1058L
12	FNA	Low	M1058L	M1058L	M1058L	–	–
	IB	Low	M1058L	M1058L	M1058L	M1058L	M1058L
13	FNA	Neg	Mixed FCoV	Neg	Neg	–	–
	IB	Low	High	Neg	Neg	Neg	Neg
14	FNA	Neg	Low	Low	Neg	–	–
	IB	Neg	Low	Neg	Neg	Low	Low
15	FNA	Neg	Neg	Neg	Neg	–	–
	IB	Neg	Low	Low	Mixed FCoV	Mixed FCoV	Non-mutated *S* gene
16	FNA	M1058L	M1058L	M1058L	M1058L	–	–
	IB	M1058L	M1058L	M1058L	M1058L	M1058L	M1058L
17	FNA	Neg	Mixed FCoV	Mixed FCoV	Mixed FCoV	–	–
	IB	Mixed FCoV	M1058L	Mixed FCoV	Mixed FCoV	Mixed FCoV	Mixed FCoV
18	FNA	Low	High	Low	Low	–	–
	IB	Low	High	High	High	High	High
19	FNA	Low	M1058L	Mixed FCoV	Mixed FCoV	–	–
	IB	Mixed FCoV	Mixed FCoV	M1058L	Mixed FCoV	Mixed FCoV	Mixed FCoV
20	FNA	Low	Low	Low	Low	–	–
	IB	Low	Low	Low	Mixed FCoV	Low	Low

* M1058L = positive *S* gene mutation RT-PCR resulting in amino acid substitution M1058L

† Neg = negative *7b* gene RT-PCR

‡ Low = positive *7b* gene RT-PCR with viral load below cut-off (therefore no further differentiation possible)

§ Mixed FCoV = feline coronavirus with and without *S* gene mutations

¶ Non-mutated *S* gene = positive *7b* gene RT-PCR but negative *S* gene RT-PCR

∞ High = positive *7b* gene RT-PCR with high viral load but no further differentiation possible

FNA = fine-needle aspirate; IB = incisional biopsy

**Table 3:** Results of *7b* gene and *spike* gene mutation RT-PCRs in different body fluids

Cat	EDTA blood	Buffy coat smear	Serum	Effusion	Peritoneal lavage	CSF	Aqueous humour
1	Low*	Low	–	Low	–	M1058L^†^	M1058L
2	M1058L	Low	Low	M1058L	–	Neg^‡^	Neg
3	–	Neg	–	–	Neg	–	Neg
4	Neg	Neg	Neg	–	Mixed FCoV^§^	Mixed FCoV	Neg
5	Low	Low	Neg	–	Neg	Low	Neg
6	M1058L	Neg	–	M1058L	–	M1058L	Neg
7	Low	Neg	Low	Mixed FCoV	–	Neg	Neg
8	Low	Low	Low	M1058L	–	Neg	Low
9	Low	-	-	M1058L	–	Low	Neg
10	Low	Low	Low	High^¶^	–	M1058L	High
11	–	–	–	Neg	–	M1058L	Neg
12	–	Neg	–	M1058L	–	–	Neg
13	Neg	–	–	–	Neg	Neg	Neg
14	–	–	Neg	Neg	–	M1058L	Neg
15	Neg	Neg	Neg	–	Neg	M1058L	Mixed FCoV
16	–	–	Neg	M1058L	–	Neg	Neg
17	–	–	–	Mixed FCoV	–	Neg	Neg
18	M1058L	Low	Neg	–	Low	High	Low
19	Low	Low	Neg	Mixed FCoV	–	–	Neg
20	–	–	–	Low	–	–	Neg

* Low = positive *7b* gene RT-PCR with viral load below cut-off (therefore no further differentiation possible)

† M1058L = positive *S* gene mutation RT-PCR resulting in amino acid substitution M1058L

‡ Neg = negative *7b* gene RT-PCR

§ Mixed FCoV = feline coronavirus with and without *S* gene mutations

¶ High = positive *7b* gene RT-PCR with high viral load but no further differentiation possible

CSF = cerebrospinal fluid

**Table 4:** Percentages of positive results of RT-PCR detecting feline coronavirus without (*7b* gene RT-PCR) and with *spike* gene mutations (*S* gene mutation RT-PCR) in different tissues and body fluids and of immunohistochemistry (IHC) in different tissues

Sample		n	*7b* gene RT-PCR (%)	*S* gene mutation RT-PCR (% [95% CI])	IHC (%)
Popliteal lymph node	FNA	20	65	15 (0–30.6)	–
	IB	20	70	30 (9.9–50.1)	NA
Mesenteric lymph node	FNA	20	85	45 (23.2–66.8)	–
	IB	20	95	45 (23.2–66.8)	80
Liver	FNA	20	85	40 (18.5–61.5)	–
	IB	20	80	40 (18.5–61.5)	60
Spleen	FNA	20	80	40 (18.5–61.5)	–
	IB	20	80	50 (28.1–71.9)	75
Omentum	IB	20	90	50 (28.1–71.9)	70
Kidneys	IB	20	85	50 (28.1–71.9)	45
EDTA blood		13	76.9	23.1 (0.2–46.0)	–
Buffy coat smear		13	53.9	0	–
Serum		11	36.4	0	–
Effusion		14	85.7	64.3 (39.2–89.4)	–
Peritoneal lavage		6	33.3	16.7 (0–46.5)	–
CSF		16	62.5	43.8 (19.4–68.1)	–
Aqueous humour		20	25	10 (0–23.1)	–

CI = confidence interval; FNA = fine-needle aspirate; IB = incisional biopsy; NA = not available; CSF = cerebrospinal fluid

The probability of finding FCoV with *S* gene mutations in an individual cat increased when specific samples were combined for analysis. Combining different organ IBs (mesenteric lymph nodes, liver, spleen, omentum, kidneys), which can be collected in a patient during laparotomy, increased the probability of finding FCoV with a mutated *S* gene to up to 80.0%. When only samples obtained by minimally invasive techniques (EDTA blood, effusion if present, fine-needle aspiration of mesenteric lymph nodes, liver, spleen) were considered, the probability of finding FCoV with mutated *S* gene increased to up to 70.0% in a patient with effusion and to up to 60.0% in a patient without effusion.

In four cats, a high FCoV load was detected by *7b* gene RT-PCR in up to seven different sample types, but no further differentiation was possible by *S* gene mutation RT-PCR; therefore, these samples were considered as negative for *S* gene mutations.

## Discussion

This study investigated the presence of FCoV with and without *S* gene mutations in different tissue and body fluid samples from cats with IHC-confirmed FIP via real-time RT-PCR.

The study was able to confirm results of previous studies, in which FCoV with mutated *S* gene were detected in effusion but not in serum or plasma from cats with FIP.^25–27^ The prevalence of FCoV with *S* gene mutations detected by RT-PCR was 64.3% in effusion, which is similar to the results of other studies (68.6% and 65.3%, respectively),^25,27^ while in one study, the prevalence was even higher (85.0%).^26^ Other fluids examined (EDTA blood, peritoneal lavage, buffy coat smears, CSF, aqueous humour) showed only low-to-moderate numbers of positive RT-PCR results for FCoV with and without *S* gene mutations. Earlier studies obtained similar results.^27,34,35^ As only 3/20 patients of this study’s population suffered from ocular or neurological symptoms, a higher prevalence of FCoV with and without *S* gene mutations might be expected in CSF or aqueous humour of patients with corresponding signs. In a previous study examining CSF, the prevalence of all FCoV detected by RT-PCR increased from 42.1% in all cats to 85.7% when considering only cats with neurological or ocular signs.^36^ In the present study, FCoV with a mutated *S* gene was detected in the CSF of both cats with neurological signs.

The study was also able to confirm previous results regarding the prevalence of the two different *S* gene mutations investigated. In the present study, only *S* gene mutation in nucleotide 23531 (resulting in amino acid substitution M1058L) was identified; *S* gene mutation in nucleotide 23537 (resulting in amino acid substitution S1060A) was not identified in any of the examined samples. Already, when those specific *S* gene mutations were detected for the first time, amino acid substitution M1058L was more common (n = 108/118) than S1060A (n = 5/118) in all examined FCoVs.^5^ Later studies confirmed these findings and only detected few^25–27^ or no FCoV at all with S1060A.^33^ As such, M1058L is the more common S protein substitution, which is also reflected by the results of the present study.

The present study detected a higher number of samples with FCoV by *7b* gene RT-PCR (detecting any FCoV) than by *S* gene mutation RT-PCR (detecting FCoV with mutated *S* gene) as only those positive in *7b* gene RT-PCR were analysed by *S* gene mutation RT-PCR. For example, *7b* gene RT-PCR was commonly positive in intra-abdominal organs (mesenteric lymph nodes, liver, spleen, kidneys, omentum; prevalence of all FCoV 80–95%). This is in accordance with other studies, in which omentum, mesenteric lymph nodes and spleen were identified as the organs with highest viral loads.^37^

In contrast, the percentage of samples positive in *S* gene mutation RT-PCR only ranged from 40% to 50% in intra-abdominal organs. One reason for this could be the presence of *S* gene mutations that remain undetected by RT-PCR because of a FCoV load below the cut-off for successful differentiation. This has already been observed in other studies using the same method.^27,33^ Another reason could be the absence of the particular *S* gene mutations examined here and the presence of other mutations involved in FIP pathogenesis instead.^6,8,9,14,15,38,39^ Some other mutations, such as in the *3c* gene, have been discussed as playing a role in FIP pathogenesis, but a clear causal relationship to FIP still has not been identified.^6,7,40,41^ Most likely, a combination of different mutations leads to the FCoV virulence change and, ultimately, to the development of FIP. As such, some of the cats in the present study might have experienced other mutations in their viral genome and therefore had negative results in *S* gene mutation RT-PCR.

Infection with serotype II FCoV could be another reason for a negative *S* gene mutation RT-PCR despite a high viral load, as *S* gene mutation RT-PCR is specific for serotype I only. Serotype II is not as common as serotype I in central European cats,^42^ but studies showed that mono-infection with serotype II occurs in cats with FIP, as does a concurrent infection with both serotypes.^43,44^ Multiple mutations in the *S* gene of serotype II FCoV that contribute to FIP development have previously been identified.^16^ Furthermore, mutations or sequence variations occurring at the primer binding site could cause negative *S* gene mutation RT-PCR results.

These reasons could explain the negative results in four cats (numbers 8, 10, 13, 18) in which FCoV load was high in some samples, but FCoV with *S* gene mutations was not detected. Interestingly, although *S* gene mutation RT-PCR was negative despite a high virus load in one sample, FCoV with *S* gene mutation or mixed FCoV (both FCoVs with and without *S* gene mutations) were detected in at least one different tissue or fluid in all of the four cats. For example, cat 18 had a high FCoV load in multiple organ samples, but *S* gene mutation RT-PCR was negative in these samples. However, FCoV with mutated *S* gene was detected in EDTA blood. This cat had histological lesions typical for FIP and positive IHC in the majority of organs, which confirms that FIP was present. These findings emphasise that a concurrent infection with different FCoV strains (non-mutated and mutated) is obviously possible and that in terms of virus kinetics, the process of evolving FIP in a patient is not a stable state.

The fact that non-mutated FCoV was detected in mesenteric lymph nodes and kidneys of cats 7 and 15 also highlights fluctuating virus kinetics. It is either possible that the non-mutated FCoV detected was circulating non-mutated FCoV that had already been present in these cats before FIP evolved or that a superinfection with non-mutated FCoV had occurred which led to systemic spread of non-mutated FCoV as described previously.^24^ Detection of mutated and non-mutated FCoV within one cat in the present study confirms that coexistence of varying FCoV strains is common within one animal. Those findings have to be considered when performing RT-PCR. A ‘negative’ result of the *S* gene mutation RT-PCR does not rule out that the cat has FIP.

Furthermore, the present study investigated which sample types (IBs, FNAs) are appropriate for virus detection. Percentages of positive RT-PCR results were similar for FNAs and IBs in most intra-abdominal organs and identical in mesenteric lymph nodes and liver for *S* gene mutation RT-PCR and in spleen for *7b* gene RT-PCR. This is an unexpected but important result, as obtaining an IB is highly invasive and usually cannot be performed without anaesthesia. An earlier study examined whether FNA and tissue biopsies taken with a needle core device of liver and kidneys would be equally useful for diagnosing FIP via immunostaining (IHC or immunocytochemistry) and, in contrast to the findings of the present study, reported that sensitivities of immunostaining in the minimally invasive FNA and tissue biopsies were not satisfactory (11–31%).^45^ In the present study, the percentage of positive *7b* gene RT-PCR results in both FNA and IB was similar to or sometimes even higher than the percentage of positive IHC in the respective organs (Table 4). This demonstrates the advantage of RT-PCR detecting small amounts of virus,^31^ whereas immunostaining requires more material and intact cells. Of course, histopathology and IHC, which are performed in combination, have the advantage of giving indicators to the presence of other disease processes and not just presence or absence of FCoV. But when only minimally invasive sampling is possible and cytology is non-diagnostic, RT-PCR should be preferred over immunostaining to detect FCoV. Another advantage of FNA is the possibility of targeting various locations; for example, ultrasound-guided sampling of several lesions or regions within organs. This is beneficial, as virus distribution can be inhomogeneous within an organ.

One limitation to this study was the fact that collection of some samples occurred post mortem. Samples collected ante mortem might have provided higher amounts of viable viral RNA. Furthermore, unclassified FCoV strains detected by *7b* gene RT-PCR (eg, in cats with high viral loads but negative *S* gene mutation RT-PCR) were not further analysed by an RNA sequencing approach, so it is unknown whether and which other mutations might have been present. Next generation sequencing of the S2 region would be very valuable in the future, in order to obtain insights into other possible mutations involved in FIP pathogenesis.

## Conclusions

FCoVs with mutated *S* genes were detected in all examined cats with FIP in at least one tissue or body fluid. Serum and buffy coat smears were the only sample types in which FCoV with mutated *S* gene was never detected. The prevalence of FCoV with a mutated *S* gene was highest in effusion. Non-mutated and mixed FCoV infections were detected in some cats, highlighting the possibility that several FCoV strains can be present within one host.

Considering FCoV detection, *7b* gene RT-PCR can be an alternative to IHC in tissues with histopathological changes consistent with FIP. In this study, it provided a higher number of positive results for FCoV than IHC. Furthermore, it can be used on samples obtained by minimally invasive techniques if tissue biopsies and thus IHC is not possible.
